# Effect of a participatory intervention in women’s self-help groups for the prevention of chronic suppurative otitis media in their children in Jumla Nepal: a cluster-randomised trial

**DOI:** 10.1186/s12887-019-1539-y

**Published:** 2019-05-23

**Authors:** Susan Clarke, Robyn Richmond, Heather Worth, Rajendra Wagle, Andrew Hayen

**Affiliations:** 1 School of Public Health and Community Medicine, University of New South Wales Sydney, High St, Kensington, NSW 2052 Australia; 2 Institute of Medicine, Tribhuvan University, Maharaganj, Kathmandu, Nepal; 3 Faculty of Health, University of Technology Sydney, 15 Broadway, Ultimo, NSW 2007 Australia

**Keywords:** Otitis media, Children, Nepal, Health promotion

## Abstract

**Background:**

Chronic suppurative otitis media (CSOM) causes preventable deafness and disproportionately affects children living in poverty. Our hypothesis was that health promotion in women’s groups would increase their knowledge, attitudes and practices (KAP) regarding ear disease and reduce the prevalence of CSOM in their children.

**Methods:**

We did a cluster randomised trial in two village development committees (VDCs) in Jumla, Nepal. In July 2014, 30 women’s groups were randomly allocated to intervention or control, stratified by VDC and distance to the road. The intervention groups participated in three sessions of health promotion using the *WHO Hearing and Ear Care Training Resource Basic Level*. The primary outcome was women’s KAP score and the secondary outcome was prevalence of CSOM in their children at 12 month follow-up. Analyses were by intention to treat. Participants and the research team were not masked to allocation.

**Results:**

In June and July 2014 we recruited 508 women and 937 of their children. 12 months later there was no difference in the women’s KAP score (mean difference 0.14, 95% CI − 0.1 to 0.38, *P* = 0.25) or the prevalence of CSOM in their children (OR 1.10, 95%CI 0.62 to 1.84, *P* = 0.75) between intervention and control groups. However, overall, there was a significant improvement in the KAP score (mean difference − 0.51, 95% CI − 0.71,to − 0.31, *P* < 0.0001) and in the prevalence of CSOM from baseline 11.2% to follow-up 7.1% (*P* < 0.0001).

**Conclusions:**

Health promotion in women’s groups did not improve maternal KAP or reduce prevalence of CSOM. Over time there was a significant improvement in women’s KAP score and reduction in the prevalence of CSOM which may be attributable to our presence in the community offering treatment to affected children, talking to their parents and providing ciprofloxacin drops to the local health posts. More research is needed in low resource settings to test our findings.

**Trial registration:**

Australia and New Zealand Clinical Trial Registry 12,614,000,231,640; Date of registration: 5.3.2014: Prospectively registered.

**Electronic supplementary material:**

The online version of this article (10.1186/s12887-019-1539-y) contains supplementary material, which is available to authorized users.

## Background

Chronic suppurative otitis media (CSOM) is a multifactorial disease of poverty. Globally, CSOM affects 65 to 330 million people, of whom at least 50% suffer clinically significant hearing loss [1]. Hearing loss can interfere with early childhood development and decrease educational and social opportunities compounding the existing disadvantage of marginalised children [2]. Therefore, effective strategies for preventing CSOM in low resource settings are urgently needed.

Otitis media is a spectrum of disease beginning with an acute respiratory infection leading to acute otitis media and otitis media with effusion, which can result in a chronic perforation of the tympanic membrane, chronic inflammation of the middle ear cavity and otorrhoea or discharge (CSOM) [1]. Appropriate treatment of acute otitis media with oral antibiotics and early treatment of CSOM with topical antibiotics and ear mopping are simple, inexpensive and effective [3]. The prevalence of CSOM in Nepal is 5 to 10% in the available studies which mostly rely on cross-sectional groups of school children in less remote settings or patients attending ear, nose and throat specialist clinics [4–7]. Every study that has been conducted in Nepal has revealed a prevalence above the 4% level satisfying the WHO definition of a ‘massive public health problem’ requiring ‘urgent attention’ [1].

Nepal is in the lower third of countries for human development (HDI 0.574, rank 149 out of 189) [8]. However, poverty remains ‘highly asymmetric’ in Nepal with the western regions and the mountains having poorer outcomes on every measure [9, 10]. Jumla is one of the most disadvantaged districts of Nepal, with an HDI of 0.409, a rank of 68 out of 75 districts [11]. CSOM is strongly associated with poverty and its social determinants including: low parental education level, low parental income, malnutrition, overcrowding, lack of access to clean water and sanitation [12, 13].

Until now, there has been little research into prevention of CSOM in low resource settings, leading to calls to have it added to the other 17 neglected tropical diseases [14]. Like the neglected tropical diseases, CSOM disproportionately affects people living in poverty causing significant morbidity, could be amenable to public health intervention and is neglected by research. A range of community based interventions have been successful in improving maternal and child health outcomes in low resource settings [15]. To our knowledge, the effectiveness of a community based educational intervention to improve the ear health of children has not previously been tested in a controlled trial. We hypothesised that in the disadvantaged mountain district of Jumla a community based intervention would improve the knowledge, attitudes and practices of women regarding ear disease and reduce the prevalence of CSOM in their children.

## Methods

### Study design

We conducted a cluster randomised trial using women’s self-help groups as the units of randomisation and individual women and their children as the units of analysis. A CRT was a suitable study design because the intervention was delivered at the cluster level and to reduce experimental contamination. The study protocol is published and we adhered to the CONSORT guidelines extension for cluster randomised trials for the study design and analysis [16, 17]. The study setting consisted of two village development committees (VDCs) in the remote mountain district of Jumla, Nepal. VDCs are the smallest local government division in Nepal and consist of 3000 to 5000 people. The pre-existing women’s self-help groups were facilitated by a local non-government organisation (NGO) which had been working in health and community development in Jumla for many years.

### Participants

The participants were women attending existing women’s self-help groups in Jumla and their household children aged 12 years and under. The only exclusion criterion was women who were unable to give informed consent. The research process was verbally explained to the participants individually and they individually gave verbal and written informed consent for themselves and their children. In addition to the parental consent, children over the age of 7 years also gave verbal and written assent as required by Nepal Health Research Council. A small donation of $US10 was contributed to each study group’s savings. Women were free to opt out of the study at any time. We aimed to enrol all group members as they would be receiving the intervention at their regular monthly meetings.

### Randomisation and masking

We randomly selected 30 women’s self-help groups from a total of 57 groups and then randomly allocated the 30 study groups to the trial arms. Randomisation was conducted by a public health officer in the district health office in Jumla, who had no other role in the research using Excel random number generator. Randomisation was stratified by VDC and distance to the road, to ensure that we included equal proportions of groups from both VDCs and from the most remote villages. All women in the study groups and their children aged 12 years and under were invited to participate by the NGO staff. Because of the pragmatic nature of the intervention, neither participants nor field-workers could be masked to study group allocation. The follow-up data was collected 12 months after the intervention.

### Procedures

The existing women’s self-help groups meet monthly to develop action plans for community problems, deposit into the group savings and participate in health education. Health education is facilitated by the NGO staff on topics such as the importance of breastfeeding, child nutrition, handwashing and safe food storage. All 30 study groups met as usual each month. The control groups received the usual education session while the intervention groups participated in additional ear health education over three consecutive group meetings. The lead author delivered the Sessions 1 and 2 with the assistance of an interpreter when needed. Session 3 was delivered by the NGO group facilitators.

Session 1 was an interactive education session in the women’s self-help groups using a flip-book containing local photographs following the sections in the *WHO Primary Ear and Hearing Care Resource Basic Level* [18]. The book focused on identification of a child with an ear infection, the causes and complications of ear infections and the consequences of hearing loss. We encouraged care-givers to attend the health post if they thought their child had an ear infection. Session 2 was a practical session consisting of hands-on ear mopping and the correct installation of eardrops, along with reinforcement of the messages from the first session. The lead author and the interpreter demonstrated on each other and then the women practised on each other. The women were very engaged in this session and actively participated, asking questions and sharing experiences. Session 3 was a brief recap of sessions one and two and included a small laminated card for each woman to take home with pictures of ear-wicking and drop installation in a child’s discharging ear.

### Outcomes

The primary outcome was the knowledge, attitudes and practices questionnaire score at 12 month follow-up assessment. The secondary outcome prevalence of childhood CSOM at the 12 month follow-up assessment. Ancillary outcomes included before and after analysis and further analysis of children’s anthropometry, socioeconomic status, caste and gender.

The primary outcome was assessed by a questionnaire that we developed since there was no existing validated tool (Additional file 1). The questionnaire was informed by the literature and includes validated questions from the demographic health survey and multiple indicator cluster survey [10, 19]. It contains demographic questions such as age, gender, number of children, maternal education, food security and usual health practices, followed by questions about knowledge, attitudes and practices regarding ear health, hearing, ear disease and healthcare seeking. The questionnaires were completed on paper in Nepali by trained research assistants.

For the secondary outcome, we used the WHO definition of CSOM as ‘a chronic inflammation of the middle ear and mastoid cavity, which presents with recurrent otorrhoea through a tympanic membrane perforation’, with at least 2 weeks of otorrhoea [1]. The lead author performed all of the ear examinations at baseline and follow-up. We collected images of tympanic membranes Cellscope-Oto smartphone enabled digital otoscope for blinded analysis. We offered a general health check to all of the children and the trained research assistants performed height, weight and visual acuity examinations. Children with ear infections were offered treatment with ciprofloxacin drops.

### Statistical analysis

Using data extracted from our initial qualitative research, the sample size was 114 women in each arm for an unclustered study with a 5% two-sided Type 1 error and 80% power to detect a 25% difference in mean knowledge, attitudes and practices scores. The cluster sizes were set at the size of the women’s groups, at around 20 women. There was no directly comparable ICC in the literature, so studies on other aspects of child health were considered in Nepal [20]. We used the safe equation DEff = 1 + (*m* − 1) ρ, assuming ρ = 0.05, which would give a DEff = 1.95, or 223 women per arm. This would translate into 11 clusters per arm. To account for clustering and loss to follow-up, a conservative 15 clusters per arm were recruited.

The primary outcome, the knowledge, attitudes and practices at follow-up assessment, was analysed using general estimating equations (GEE), which adjust for clustering because groups rather than individuals were randomised. Covariates from the literature including socioeconomic status, caste, parental education and nutritional status were also considered using GEE. The secondary outcome, the prevalence of CSOM at follow-up assessment, was similarly analysed using GEE.

We also performed several ancillary analyses. Further analysis of a comparison of baseline and follow-up data was carried out using standard statistical techniques, including simple *t*-tests for the continuous knowledge, attitudes and practice data and McNemar’s test for our binary data. Similarly, several important correlates were examined individually using similar standard techniques. Analysis was by intention-to-treat using SPSS version 25. Since there were no potential harms from the intervention there was no data monitoring committee.

## Results

**Fig. 1:**
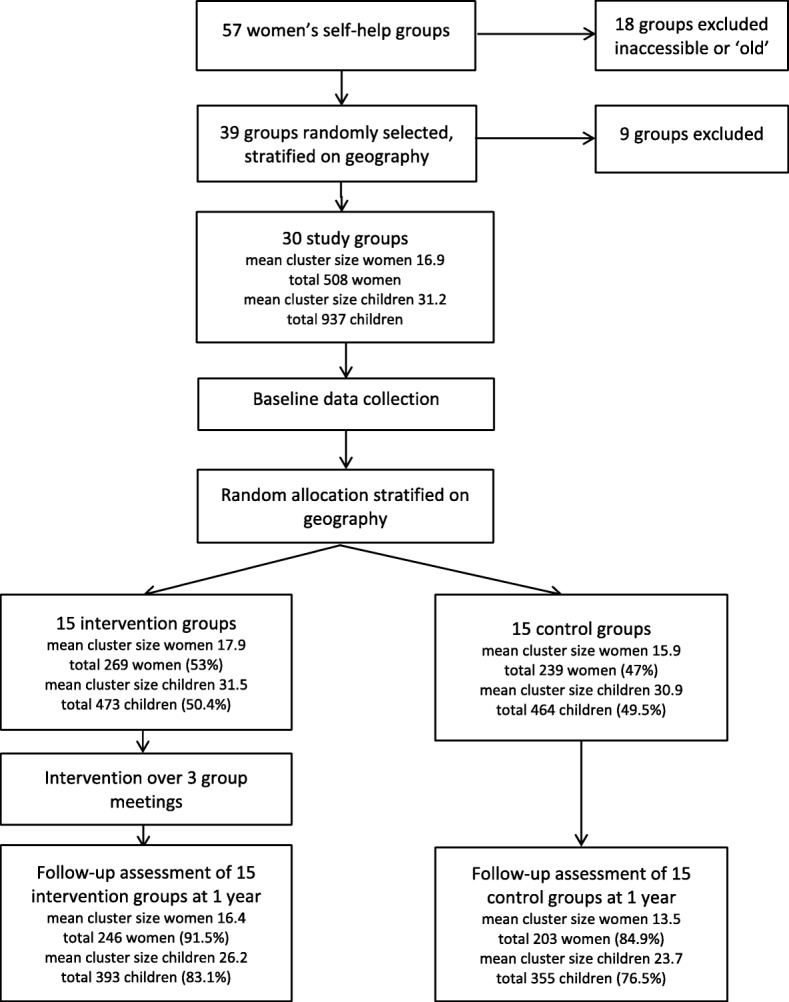
Flow of participants through the trial

We recruited 30 groups, which comprised 508 women and 937 of their children between Jun 1, 2014 and Jul 31, 2014. Figure 1 presents the cluster and individual participant flow. Follow-up assessment was performed on 449 (88.4%) of the women and 748 (79.8%) of their children.

Table 1 shows the baseline characteristics of the women and children. The mean age of the women was 34.3 (SD 11.3) years, they had 3.4 children (SD 1.6) and more than half (52.8%) of the women had received no education. Intervention and control groups were similar on all measures except for caste. More women in the intervention groups belonged to Dalit caste (115, 42.8%) compared to control group (58, 24.3%). Women in 22 of the 30 clusters belonged to a single type of caste, either Brahman/Chhetri or Dalit, while three groups had one or two other caste members and two other groups had four or five. The completely Brahman/Chhetri groups were equally distributed in the intervention and control groups (eight in the intervention and nine in the control) but the completely Dalit groups were not (six in the intervention and two in the control). Therefore, since randomisation was performed by group, there were more women of Dalit caste in the intervention group than in the control group. Table 2 shows the baseline characteristics of the clusters. The intervention and control clusters were similar on all measures except for caste. Intervention groups had a mean of 7.7 Dalit women per group and control groups had a mean of 4.8 women per group.

**Table 1:** Baseline characteristics of women and children

	Intervention	Control	Total
Number of women (%)*	269 (53)	239 (47)	508
Number of clusters	15	15	30
Cluster size (mean, SD)	17.9 (2.3)	15.9 (3.3)	16.9 (3.0)
Age of women (mean, SD)	33.8 (11.5)	34.9 (11.0)	34.3 (11.3)
Number of children per woman (mean, SD)	3.3 (2.3)	3.5 (1.6)	3.4 (1.6)
Women’s caste (%)			
Dalit	115 (42.8)	58 (24.3)	173 (34.1)
Brahman/Chhetri	154 (57.2)	181 (75.7)	335 (65.9)
Household size (mean, SD)	6.1 (2.6)	6.1 (2.6)	6.1 (2.6
Area household land in halsϮ (mean, SD)	3.3 (2.3)	3.6 (3.5)	3.4 (3.0)
Number of household cattle and buffalo (mean, SD)	3.6 (2.5)	3.8 (2.9)	3.7 (2.7)
Any smoking inside the house (%)	151 (56.1)	120 (50.2)	271 (47.1)
Highest education level women (%)			
None	232 (86.2)	205 (85.8)	437 (86)
Some primary	21 (7.8)	15 (6.3)	36 (7.1)
Some secondary	16 (5.8)	19 (7.9)	35 (6.9)
Highest education level husband (%)			
None	149 (55.4)	119 (49.8)	268 (52.8)
Some primary	46 (17.1)	55 (23)	101 (19.9)
Some secondary	74 (27.5)	65 (27.3)	139 (27.4)
Any antenatal care last pregnancy (%)	238 (88.5)	220 (92.1)	458 (90.2)
Location of last birth (%)			
In the cowshed	72 (26.8)	71 (29.7)	143 (28.1)
Inside the house	126 (46.9)	113 (47.3)	239 (47.1)
At a health facility	62 (23.1)	49 (20.5)	111 (21.8)
Ever taken a child to traditional healer (%)	124 (46.1)	109 (45.6)	233 (45.9)
Number of participant children (%)			
Total	473 (50.5)	464 (49.5)	937
Girls	241 (51)	229.(49.4)	470 (49.8)
Boys	232 (49)	235 (50.6)	467 (50.2)
Age of children (mean, SD)	6.5 (3.5)	6.5 (3.5)	6.5 (3.5)
Children’s anthropometry (mean, SD)			
Weight of children in kg	17.31 (6.95)	17.72 (7.19)	17.51 (7.07)
Weight-for-age *z*-score	−1.86 (1.07)	−1.88 (1.04)	−1.9 (1.05)
Height of children in cm	106.47 (21.21)	108.03 (21.95)	107.22 (21.57)
Height-for-age *z*-score	−2.05 (1.36)	−1.97 (1.28)	−2.0 (1.33)
Children’s ear examination (%)			
Any CSOM	53 (11.2)	53 (11.4)	106 (11.3)
Any acute otitis media	5 (1.1)	13 (2.8)	18 (1.9)
Any dullness or retraction eardrum	37 (7.8)	67 (14.4)	104 (11.2)

*Data are number (%) or mean (SD) as indicated

**Table 2:** Baseline characteristics of clusters (women’s self-help groups)

	Intervention	Control	Total
Number of clusters (women’s self-help groups)	15	15	30
Mean number of women in clusters (SD)	17.93 (2.25)	15.93 (3.28)	16.93 (2.97)
Number of clusters in each VDC			
VDC 1	5	5	10
VDC 2	10	10	20
Numbers of clusters at distance to the health post (N)			
Less than one hour	12	10	22
One hour or more	3	5	8
Mean age of women in clusters (mean, SD)	33.58 (3.48)	35.15 (2.03)	34.36 (2.91)
Number of children per woman (mean, SD)			
Total	3.26 (0.39)	3.47 (0.39)	3.37 (0.49)
Girls	1.76 (0.38)	1.81 (0.24)	1.79 (0.32)
Boys	1.52 (0.29)	1.66 (0.36)	1.59 (0.33)
Mean number of women of Dalit caste per cluster (SD)	7.67 (8.04)	4.80 (5.74)	6.23 (7.02)
Household size (mean, SD)	6.06 (0.91)	6.15 (0.90)	6.10 (0.89)
Area of household land in hals (mean, SD)	3.29 (1.03)	3.39 (1.47)	3.34 (1.25)
Number of household cattle and buffalo (mean, SD)	3.72 (1.23)	3.73 (1.36)	3.72 (1.22)
Mean number of households with indoor smoking per cluster (SD)	9.80 (3.41)	7.80 (3.47)	8.80 (3.53)
Highest education level woman (mean, SD)			
None	15.47 (3.16)	13.67 (2.77)	14.57 (3.06)
Some primary	1.47 (1.13)	1.20 (1.27)	1.33 (1.18)
Some secondary	1.0 (1.60)	1.07 (1.53)	1.03 (1.54)
Highest education level husband (mean, SD)			
None	9.93 (4.52)	7.93 (3.60)	8.93 (4.14)
Some primary	3.13 (1.81)	3.67 (2.09)	3.40 (1.94)
Some secondary	4.93 (4.10)	4.33 (3.48)	4.63 (3.75)
Any antenatal care last pregnancy (mean, SD)	15.87 (3.09)	14.67 (3.66)	15.27 (3.38)
Location of last birth (mean, SD)			
In the cowshed	4.80 (3.73)	4.73 (3.08)	4.77 (3.36)
Inside the house	7.53 (4.94)	7.53 (4.94)	7.97 (4.85)
At a health facility	4.20 (2.54)	3.80 (2.40)	4.0 (2.44)
Ever taken child to traditional healer (mean, SD)	8.27 (3.58)	7.27 (4.54)	7.77 (4.05)
Mean number of participant children per cluster (SD)			
Total	31.53 (9.23)	30.93 (9.85)	31.23 (9.39)
Girls	16.07 (6.49)	15.27 (6.11)	15.67 (6.21)
Boys	15.47 (4.75)	15.67 (5.65)	15.57 (5.13)
Age of children (mean, SD)	6.48 (1.05)	6.49 (1.05)	6.49 (0.96)
Children’s anthropometry (mean, SD)			
Weight of children in kg	17.28 (1.87)	17.23 (2.01)	17.26 (1.91)
Weight-for-age *z*-score	−1.96 (0.33)	−1.93 (0.33)	− 1.95 (0.33)
Height of children in cm	106.51 (6.16)	107.50 (6.01)	107.0 (6.0)
Height-for-age *z*-score	−2.09 (0.41)	−1.98 (0.34)	− 2.03 (0.37)
Children’s ear examination (mean, SD)			
Any CSOM	3.67 (1.76)	3.40 (2.06)	3.53 (1.89)
Any acute otitis media	0.33 (0.62)	0.87 (1.06)	0.6 (0.89)
Any dullness or retraction eardrum	2.67 (1.95)	4.27 (3.20)	3.47 (2.73)

We analysed the primary outcome at both the cluster and individual level (see Table 3). The main analysis using GEE and the null model gave non-significant results (mean difference = 0.14, 95% CI − 0.10 to 0.38, *P* = 0.25), as did the model that included geographical stratification (mean difference = 0.15, 95% CI − 0.09 to 0.38, *P* = 0.21). VDC 1 consistently had KAP lower scores in this model (mean difference = − 0.78, 95% CI − 1.0 to − 0.55, *P* < 0.0001). The ICC was 0.14, indicating a large degree of clustering.

**Table 3:** Comparison of women’s KAP scores at 12 month follow-up in the intervention and control groups, using cluster-level summaries and individual-level regression analysis

	Unadjusted analysis	Adjusted analysis^a^
Cluster-level analysis		
Mean difference	0.03	0.06
95% CI	−0.41 to 0.47	0.41 to 0.30
*P* value	0.88	0.75
Linear regression unadjusted for clustering		
Mean difference	−0.14	− 0.15
95% CI	−0.39 to 0.10	− 0.39 to 0.09
*P* value	0.26	0.23
Mixed effects linear regression		
Mean difference	0.12	0.14
95% CI	−0.36 to 0.61	−0.28 to 0.56
*P* value	0.61	0.49
Generalised estimating equations		
Mean difference	0.14	0.15
95% CI	−0.10 to 0.38	−0.09 to 0.38
*P* value	0.25	0.21

^a^adjusted for VDC and distance from the road

Table 4 shows the GEE analysis of the covariates of the primary outcome demonstrating no significant difference between the KAP score in the intervention or control groups (mean difference = 0.14, 95% CI − 0.10 to 0.38, *P* = 0.27). Women of Dalit caste, who lived in VDC 2, and those with a larger number of children and number in household were all associated with a higher KAP score. Measures of socioeconomic status (amount of land and number of large animals owned) were not associated with the outcome; nor were smoking inside or the level of education reached by the woman or her husband.

**Table 4:** Covariates of women’s KAP score at 12 month follow-up in the intervention and control groups, using generalised estimating equation (GEE)

Parameter estimates						
Parameter	B	SE	95% Wald CI	Hypothesis test		
				Wald chi-square	df	*P*
(Intercept)	7.583	0.3248	6.947 to 8.220	545.114	1	0.001
Group (control vs intervention)	137	0.1230	−0.104 to 0.378	1.241	1	0.265
VDC (1 vs 2)	−0.868	0.1301	−1.123 to −0.613	44.490	1	0.001
Distance to health post (< 1 h vs ≥ 1 h)	0.110	0.1455	− 0.175 to 0.395	0.571	1	0.450
Caste (Dalit vs Brahmin/Chhetri)	0.491	0.1611	0.175 to 0.806	9.271	1	0.002
Woman education (none vs some)	−0.145	0.1882	−0.514 to 0.224	0.593	1	0.441
Husband education (none vs some)	−0.040	0.1471	−0.328 to 0.248	0.074	1	0.786
Smoking inside (infrequently/never vs daily)	−0.142	0.1344	−0.405 to 0.122	1.113	1	0.291
Age of woman	−0.012	0.0066	−0.025 to 0.001	3.365	1	0.067
No. children per woman	0.175	0.0597	0.058 to 0.292	8.620	1	0.003
No. in household	0.059	0.0294	0.001 to 0.116	3.994	1	0.046
Household land (hals)	−0.021	0.0216	−0.063 to 0.022	0.926	1	0.336
No. cattle, buffalo, horses	−0.034	0.0231	−0.079 to 0.012	2.127	1	0.145

The secondary outcome was prevalence of CSOM in the children at 12-month follow-up. Overall, 53 (7.1%) out of 748 children were suffering CSOM at follow-up assessment, 29 (7.4%) in the intervention group and 24 (6.8%) in the control group. There were 37 children with unilateral and 16 children with bilateral CSOM, and the prevalence increased with age. Forty of the children had experienced discharge for more than 12 weeks and 29 had done so for one year or more, and there was no difference in the mean duration of discharge between the intervention and control groups. (mean difference 2.83 weeks, 95% CI 62.52 to 68.19, *P* = 0.931). Table 5 shows the analysis of the secondary outcome. The unadjusted GEE showed OR 1.10 (95% CI 0.62 to 1.92, *P* = 0.75). When adjusted for stratification the GEE model produces an OR of 1.12 (95% CI 0.64 to 1.96, *P* = 0.70). The ICC for the secondary binomial outcome was 0.06.

**Table 5:** Comparison of the prevalence of CSOM in children in the intervention and control groups at 12 month follow-up using cluster-level summaries and individual-level regression analyses

	Unadjusted analysis	Adjusted analysis^a^
Cluster-level summary analysis		
Mean difference	−0.33	0.32
95% CI	−1.41 to 0.75	−0.82 to 1.45
*P* value	0.53	0.57
Logistic regression unadjusted for clustering		
Odds ratio	1.10	1.12
95% CI	0.63 to 1.92	0.63 to 1.98
*P* value	0.75	0.71
Logistic regression with random effects		
Odds ratio	1.07	1.09
95% CI	0.62 to 1.84	0.63 to 1.89
*P*-value	0.80	0.76
Generalised estimating equations		
Odds ratio	1.10	1.12
95% CI	0.62 to 1.92	0.64 to 1.96
*P*-value	0.75	0.70

^a^adjusted for VDC and distance from the road

Next, we analysed the secondary outcome covariates using GEE (see Table 6). The null model includes the variables of geographical stratification VDC and distance to the health post, as well as group type. In the null model there was no difference between the intervention and control groups in the prevalence of CSOM at follow-up, OR 1.12, 95% CI 0.64 to 1.96, *P* = 0.76 and for the model overall χ^2^ (1*, n* = 748) = 0.15, *P =* 0.70. BMI-for-age *z-*score delivered an OR of 0.52, 95% CI 0.34 to 0.79, *P* = 0.003, and was the largest predictor in this model. The number of large animals was statistically significant but the odds ratio was very close to 1, which is a very small predictor in this model. Other measures of socioeconomic status, caste, education, smoking or geography did not make a unique contribution to the model.

**Table 6:** Covariates of prevalence of CSOM at 12 month follow-up in the intervention and control groups using generalised estimating equation (GEE)

Parameter	B	SE	Hypothesis test			Odds ratio	95% CI
			Wald chi-square	df	*P*		
(Intercept)	−4.72	1.19	15.68	1	0.001	0.009	0.001 to 0.09
Group (control vs intervention)	0.30	0.46	0.42	1	0.52	1.345	0.55 to 3.28
VDC (1 vs 2)	0.58	0.48	1.41	1	0.23	1.78	0.69 to 4.59
Distance to health post (< 1 h vs ≥ 1 h)	0.45	0.57	0.63	1	0.43	1.57	0.52 to 4.75
Caste (Dalit vs Brahmin/Chhetri)	0.21	0.53	0.16	1	0.69	1.24	0.44 to 3.49
Woman’s education (none vs some)	0.63	0.77	0.66	1	0.42	1.87	0.41 to 8.50
Area of household land (hals)	−0.24	0.14	2.78	1	0.10	0.79	0.60 to 1.04
Number of cattle, buffalo, horses	0.16	0.07	4.43	1	0.04	1.17	1.01 to 1.35
Woman’s age	−0.01	0.06	0.01	1	0.91	0.99	0.88 to 1.12
Children’s BMI-for-age *z* score	−0.66	0.22	9.07	1	0.003	0.52	0.34 to 0.79

We then compared the overall baseline and follow-up results. Firstly, for the primary outcome, we compared the mean of individual women’s baseline KAP score with the mean of their follow-up KAP scores using the paired samples *t*-test. The overall follow-up mean (mean *=* 7.72*,* SE = 0.48) was significantly greater than the overall baseline mean (mean = 7.21, SE = 0.08) KAP score (mean difference = − 0.51, 95% CI − 0.71 to − 0.31, *t* (446) = − 5.07, *P* < 0.0001). Secondly, we compared the overall baseline and follow-up prevalence of CSOM in the children. There were 106 (11.3%, *n* = 937) cases of CSOM in the baseline examination and 53 (7.1*%, n* = 749) in the follow-up examination. Using McNemar’s test to compare two related categorical variables, there was a significant reduction in the overall prevalence of CSOM at follow-up (*P* < 0.0001).

## Discussion

To our knowledge, this is the first cluster randomised trial to assess a community based intervention to prevent CSOM in a low to middle income country. In Jumla Nepal, health promotion in existing women’s self-help groups did not increase the women’s KAP for their children’s ear health or reduce the prevalence of CSOM in the children. However, there was significant overall improvement in KAP score and reduction in the prevalence of CSOM that was equal in both the intervention and control groups at 12 month followup. Our trial was powered to detect small effects and the intervention was delivered as planned so it is likely that the lack of increase in KAP and reduction of CSOM in the intervention group was a true null effect. Therefore, either our hypothesis that our health promotion would improve KAP and reduce CSOM was flawed or there were other confounding conditions.

Despite the null result from the intervention, there was a significant overall small increase in the women’s KAP score and a large decrease in the children’s prevalence of CSOM, from 11.3 to 7.1%. Although we cannot ascribe causality to this result with certainty the control group did receive an informal intervention by participating in the trial itself. The global research emphasises the difficulty of reducing CSOM, so it is most unlikely that a relative risk reduction of 37% in 1 year is a natural improvement [21]. The control group met the team, answered the survey questions twice in a 12-month period and allowed us to examine their children. When we found a child with any kind of ear disease we spoke to their parents, explained the disease, discussed treatment in detail and either gave them ciprofloxacin eardrops or referred them to the health post for oral antibiotics. There is evidence that just being asked about your behaviour can change it, a phenomenon called ‘mere measurement’, which may have affected women in the control group and this attribution effect has been found in many studies. In addition, the effect of this ‘much better than usual care’ might have been so powerful that it obscured the potential effect of the formal intervention [22].

Our study had important strengths. Our study was set among remote village women and children with a high burden of disease who are under-served by research. Our intervention was embedded in the local community, low-cost and easily reproducible in many contexts. We had a high follow-up rate and consistency in the delivery of the intervention. There was a significant equal increase in KAP scores and reduction in the prevalence CSOM in the both study groups suggesting that this was a genuine effect. Our study also had limitations. Our participants were unable to be blinded as to group allocation and our presence in the community and interest in ears was widely known. Some clusters were very near and could have contaminated the outcomes. We offered treatment to any child who presented with CSOM at any time during the study and referred any with acute otitis media to health services which, although ethically correct, potentially contaminated our findings.

The global research on prevention of CSOM is scanty, despite its morbidity and occasional mortality among disadvantaged people. Like our study, several promising interventions have not been able to demonstrate their effectiveness. CSOM is a complex condition that reflects the interaction of marginalisation, poverty, malnutrition, quality of health services, access to education and the inequity of health research. One reported trial tested the ‘Breathing, blowing, coughing’ exercise to clear mucus at the beginning of the school day which continues to be used in schools in remote Australian Aboriginal communities. Teachers reported ‘less snot’ there was no reduction in CSOM [23]. Similarly, the introduction of community swimming pools was hypothesised to reduce CSOM by passive ear toilet, but studies have shown no effect on the prevalence of CSOM [24]. Zinc supplementation has been unsuccessful [25] and even the screening program for Aboriginal children in New South Wales, Australia, has not provided evidence of a reduction in the prevalence of CSOM [26]. The failure of these studies to reduce the prevalence of CSOM demonstrates the difficulty of research into and management of CSOM. Therefore, our overall relative reduction of 37% is both meaningful and unique.

There are two interventions for the prevention of CSOM which have been successful and both use medication, so are very different to our community based intervention. In Nepal, Vitamin A was given to pre-schoolers for the prevention of blindness, and a sample were followed into adulthood. Schmitz et al. (2012) [27] found that malnourished pre-schoolers with discharging ears who were given Vitamin A had a 42% reduction in hearing loss in adulthood. The mechanism of this effect is not understood and all children in Nepal receive Vitamin A. The second intervention that has been shown to possibly reduce CSOM is pneumococcal immunisation which continues to be evaluated, with current reductions in acute otitis media of 6 to 43% reported from developed nations [28]. In addition, successful clinical treatment programs such as the Earbus in Western Australia report significant reductions in CSOM but need community engagement, skilled staff and intensive follow-up which is difficult to achieve in low resource settings such as Jumla [29].

The baseline prevalence of CSOM in our study (11.3%) was higher than in other studies in children in Nepal [4–7]. However, there are no other recent studies measuring the prevalence of CSOM in children in similarly remote and disadvantaged places in Nepal. Similar to the Nepal studies, the prevalence of CSOM children in Bangladesh and India is 3 to 6%, while Indigenous children in remote Australia and Greenland have the highest rates of CSOM, 8 to 17% [30]. Therefore, the baseline prevalence in our study population was very high on world standards, probably explained by the degree of poverty and remoteness of Jumla.

Future research might explore adapting our materials to assess maternal knowledge, attitudes and practices at earlier time points to assess whether knowledge degraded over time. Local primary health service providers could be included in the study and usage could also be assessed to triangulate practice data. In addition, since CSOM is a chronic variable condition, longer term followup and a focus on younger children would be useful.

## Conclusions

There was a significant improvement in the women’s KAP for ear health and a significant reduction in CSOM in their children at 12 months, equal in both study groups. We were not able to reject the null hypothesis that the intervention based on *WHO Primary Ear and Hearing Care Resource* [18] would improve outcomes. Our ‘contamination’ of the control group in ethically examining and treating children with CSOM, talking to their parents, stocking the local health post, this ‘much better than usual care’ may have contributed to the overall reduction in prevalence of CSOM, obscuring the effect of the formal intervention [22]. More research is urgently needed in low-resource setting to prevent the life-changing hearing loss of this neglected disease of disadvantage.

## Additional file

Additional file 1: Clarke et al., Jumla CSOM questionnaire. Original English questionnaire developed by Clarke et al., then translated into Nepali for use in Jumla, Nepal. (DOCX 25 kb)

## Abbreviations

CSOM: Chronic suppurative otitis media
GEE: General estimating eqs.
HDI: Human development index
KAP: Knowledge, attitudes and practices
NGO: Non-government organisation
VDC: Village development committee
WHO: World Health Organisation
