# Supplementary material for: Effect of a participatory intervention in women’s self-help groups for the prevention of chronic suppurative otitis media in their children in Jumla Nepal: a cluster-randomised trial
Source: BMC Pediatr. 2019 May 23;19:163. doi: 10.1186/s12887-019-1539-y (PMC6533658; doi:10.1186/s12887-019-1539-y)
Supplement: Supplementary file 1 — Clarke et al., Jumla CSOM questionnaire. Original English questionnaire developed by Clarke et al., then translated into Nepali for use in Jumla, Nepal. (DOCX 25 kb) [file 12887_2019_1539_MOESM1_ESM.docx]

ID#:

VDC:

GROUP:

1. How old are you? ___________

2. What is your caste/ethnicity?_____________

3. Did you go to school? Yes No

If Yes, what was the highest grade you reached? _____

4. Are you married? Yes No

If Yes, did your husband go to school? Yes No

If Yes, what was the highest grade he reached?_______

5. Do you have children? Yes No

If yes, how many sons? ______ How many daughters?_______

6. How many people live in your house?__________

Do your husband’s mother or father live in your house? Yes No

7. Do you or someone living in your household own the dwelling or is it rented or have other

arrangements? Own Rent

8. Does any member of your household own any land that can be used for agriculture? Yes No

If yes, how much area of agricultural land do members of your household own?

_____________________Ropani(ropani/aana/paisa)

______________________Bigha (bigha/katha/dhur)

9. Does this household own any livestock, herds, other farm animals or poultry? Yes No

If yes, how many does this household have?

Cow/Ox­­­­­­­__________

Yak/Nak/Chauri__________

Male/Female Buffalo_________

Goats/Tibetan Goats__________

Sheep_______________

Pigs/Swines___________

Horse/Asses/Mules___________

Chicken/Ducks/Pigeons_________

Other animals__________

10. Is your cooking usually done in the same house, in a separate building or outdoors?

In the house *If in the house*, is a *separate room used as a kitchen?*

In a separate room used as kitchen

Elsewhere in the house

In a separate building

Outdoors

Other (specify)

11. How often does anyone smoke inside your house? Would you say daily, weekly, monthly, less

than, monthly, or never?

Daily_____ Weekly____ Monthly____Less than monthly ____ Never ___

12. Please mention all the occasion when it is important to wash your hands.

*Circle all mentioned*

Before eating

After eating

Before praying

Before breastfeeding or feeding a child

After defecation/urination

After cleaning a child that has defecated/changing child’s nappy

When the hands are dirty

After cleaning toilet or potty

Others (specify)

Don’t know

13. Did your household have to adopt the following to meet the household food need in the last 12 months?

Take loan Yes No

Collect wild food? Yes No

Consume seed stock for next season? Yes No

Sell household assets? Yes No

Sell livestock? Yes No

Sell land? Yes No

*Probe: Any other steps taken? If yes, specify* ___________________

14. If ever given birth,

Did you see anyone for antenatal check-up during your last pregnancy?

Yes No

Where did you give birth to your last child?

Home

Own home

Other’s house

If in home, in which part? Inside____in the cowshed_____Other____

Health Post

Hospital in Jumla

Other_____________________

KNOWLEDGE QUESTIONS

*For all questions, probe anything else? Circle all, but do not prompt.*

15. Do you know any signs that a child might have an ear infection?

Ear pain____Lump behind ear___Fever___Ear Discharge___Pulling ear___Irritability____

Other________

16. Do you know any causes of ear infections in children?

Upper respiratory infections Poor hygiene

Germs Smoking

Bathing in dirty water Overcrowding

Soap going in ear Malnutrition

Family history Indoor cooking smoke

Milk going in ear Other_____________

17. Do you know any bad things that can happen to a child with ear infections?

Deafness___Abscess___Death____Other____________

18. Can you please tell me all of causes of deafness in children that you have heard?

Ear infections___Noise___Trauma___Congenital___Other infection___Other___________

19. What are the home remedies for earache or ear discharge in your village?

Oil___Sindhu___Root from jungle___ Other___________

20. How can we take care of our children’s ears?

Personal hygiene Do not swim or wash in dirty water

Do not put anything in ears Other_________________

ATTITUTE QUESTIONS

21. Do you think that runny ears are a normal part of childhood ?

Strongly agree, slightly agree, slightly disagree, strongly disagree

22. Do you agree that treatment for ear infections works well?

Strongly agree, slightly agree, slightly disagree, strongly disagree

23. How confident are you about receiving good treatment at the health post?

Extremely confident, quite confident, slightly confident, not at all confident.

24. How seriously ill does your child need to be for you to take them to the health post?

Extremely serious, quite serious, slightly serious, not at all serious.

25. How serious do you think that ear pain is?

Extremely serious, quite serious, slightly serious, not at all serious.

How serious do you think that ear discharge is?

Extremely serious, quite serious, slightly serious, not at all serious

26. Do you think that children with frequent ear infections can have trouble hearing and learning to talk? Yes___ No___ Don’t know___

PRACTICE QUESTIONS

27. Have you ever put anything in your children’s ears? Yes No

If yes, what? Oil ___Ear drops from health post/hospital____Ear drops from elsewhere (specify)____________Other________

28. Have you ever cleaned your children’s ears? Yes No

If yes, what with?_________

29. Sometimes children have severe illness and should be taken to a health facility. What types of symptoms would cause you to take your child to a health facility right away?

Child not able to drink or breastfeed *Probe, any other symptoms?*

Child becomes sicker *Keep asking for more signs or symptoms until*

Child develops a fever *mother cannot recall any more*

Child has fast breathing *Circle all but do not prompt*

Child has difficult breathing

Child has blood in stool

Child is drinking poorly

Other____________

30. Has any child in your household ever had earache ? Yes No

If Yes, tell me about the last time, did you seek any treatment or advice? Yes No

If yes, from where did you seek advice or treatment?

Hospital

INF clinic

Health Post *Probe, anywhere else*

Village Health Worker *Circle all, but do not prompt*

FCHV

Private pharmacy

Relative/friend

Shop

Home remedy

Dhami Jhakri

Other______

Where did you first seek treatment?___________________

If no, would you seek treatment or advice? Yes, no

If yes, from where would you seek advice or treatment?

Hospital

INF clinic

Health Post *Probe, anywhere else*

Village Health Worker *Circle all, but do not prompt*

FCHV

Private pharmacy

Relative/friend

Shop

Home remedy

Dhami Jhakri

Other______

Where would you first seek treatment?___________________

31. Has any child in your household ever had discharge from the ear ? Yes No

If Yes, tell me about the last time, did you seek any treatment or advice? Yes No

If yes, from where did you seek advice or treatment?

Hospital

INF clinic

Health Post *Probe, anywhere else*

Village Health Worker *Circle all, but do not prompt*

FCHV

Private pharmacy

Relative/friend

Shop

Home remedy

Dhami Jhakri

Other______

Where did you first seek treatment?___________________

If no, would you seek treatment or advice? Yes, no

If yes, from where would you seek advice or treatment?

Hospital

INF clinic

Health Post *Probe, anywhere else*

Village Health Worker *Circle all, but do not prompt*

FCHV

Private pharmacy

Relative/friend

Shop

Home remedy

Dhami Jhakri

Other______

Where would you first seek treatment?___________________

32. In the last 12 months have you visited a health facility for care of yourself or your children? Yes, No

If yes, where did you go?

Hospital *Probe, anywhere else*

INF clinic *Circle all, but do not prompt*

Health Post

Private pharmacy

Other________________

33. Have you ever taken a child in your household to the dhami jhakri for any reason? Yes No

If yes, tell me about the last time, what was the reason?______________
